# Methylome Dynamics of Bovine Gametes and *in vivo* Early Embryos

**DOI:** 10.3389/fgene.2019.00512

**Published:** 2019-05-28

**Authors:** Jingyue Ellie Duan, Zongliang Carl Jiang, Fahad Alqahtani, Ion Mandoiu, Hong Dong, Xinbao Zheng, Sadie L. Marjani, Jingbo Chen, Xiuchun Cindy Tian

**Affiliations:** ^1^Department of Animal Science, University of Connecticut, Storrs, CT, United States; ^2^School of Animal Science, AgCenter, Louisiana State University, Baton Rouge, LA, United States; ^3^Department of Computer Science and Engineering, University of Connecticut, Storrs, CT, United States; ^4^Institute of Animal Science, Xinjiang Academy of Animal Sciences, Ürümqi, China; ^5^Department of Biology, Central Connecticut State University, New Britain, CT, United States

**Keywords:** DNA methylation, gametes, single early embryo, WGBS, bovine

## Abstract

DNA methylation undergoes drastic fluctuation during early mammalian embryogenesis. The dynamics of global DNA methylation in bovine embryos, however, have mostly been studied by immunostaining. We adopted the whole genome bisulfite sequencing (WGBS) method to characterize stage-specific genome-wide DNA methylation in bovine sperm, immature oocytes, oocytes matured *in vivo* and *in vitro*, as well as *in vivo* developed single embryos at the 2-, 4-, 8-, and 16-cell stages. We found that the major wave of genome-wide DNA demethylation was complete by the 8-cell stage when *de novo* methylation became prominent. Sperm and oocytes were differentially methylated in numerous regions (DMRs), which were primarily intergenic, suggesting that these non-coding regions may play important roles in gamete specification. DMRs were also identified between *in vivo* and *in vitro* matured oocytes, suggesting environmental effects on epigenetic modifications. In addition, virtually no (less than 1.5%) DNA methylation was found in mitochondrial DNA. Finally, by using RNA-seq data generated from embryos at the same developmental stages, we revealed a weak inverse correlation between gene expression and promoter methylation. This comprehensive analysis provides insight into the critical features of the bovine embryo methylome, and serves as an important reference for embryos produced *in vitro*, such as by *in vitro* fertilization and cloning. Lastly, these data can also provide a model for the epigenetic dynamics in human early embryos.

## Introduction

Cytosine methylation plays essential roles in mammalian development, including gene expression, transposon silencing, cell differentiation, genomic imprinting, and X chromosome inactivation (Hackett and Surani, 2013). DNA methylation is relatively stable in differentiated somatic cells, but highly dynamic during primordial germ cell development and pre-implantation embryogenesis (Saadeh and Schulz, 2014). Embryonic DNA methylation reprogramming requires genome-wide DNA demethylation, which erases the epigenetic marks of the parental genomes; this is followed by rapid *de novo* methylation to establish the epigenetic state of the early embryo (Seisenberger et al., 2012). With the recent advancement of genome-wide bisulfite sequencing, several methylome studies have been conducted on mammalian pre-implantation embryos revealing new insights into the nuances of this dynamic process (Smith et al., 2012; Guo et al., 2014; Gao et al., 2017; Jiang et al., 2018; Zhu et al., 2018). For example, in the mouse, reduced representation bisulfite sequencing (RRBS) revealed rapid genome-wide demethylation in zygotes (Smith et al., 2012). In primates, however, this major demethylation event did not occur until the 2-cell stage (Guo et al., 2014; Gao et al., 2017; Zhu et al., 2018). Contrary to observations generated by immunostaining, which showed the highest overall level of DNA methylation in mouse blastocysts (Dean et al., 2001), the lowest averaged DNA methylation was found at this stage, despite the fact that *de novo* methylation had been initiated earlier (Smith et al., 2012).

Cattle are one of the most economically valuable livestock species (Woolliams, 1996). Many studies have been conducted on the global methylation dynamics of bovine embryos by immunostaining of 5 mC (Dean et al., 2001; Beaujean et al., 2004; Park et al., 2007). While immunostaining provides important overall methylation dynamics, it does not provide specific sequence information of the methylated/de-methylated regions. More recently, sequence-based approach such as EmbryoGENE DNA Methylation Array was developed to profile methylome in bovine embryos (Salilew-Wondim et al., 2015; O’Doherty et al., 2018). Although considerably more specific than immunostaining, microarrays are limited by the finite number of probes used in their construction. We were the first to report methylome dynamics at the single-base resolution in bovine *in vivo* pre-implantation embryos using a high-throughput sequencing method, RRBS (Jiang et al., 2018). However, RRBS preferentially selects CpG-rich regions, such as CpG islands, while CpG shores are usually under-represented (Doherty and Couldrey, 2014). These shore regions are known to play important roles in tissue differentiation (Doi et al., 2009). Recently, the development of single-cell whole genome bisulfite sequencing (WGBS) allowed for the reliable and affordable revelation of potentially all CpG sites in a single oocyte or embryo (Smallwood et al., 2014). Thus, we sought to complement our previous work by analyzing bovine gametes and *in vivo* pre-implantation embryos using WGBS. These data will provide the gold standard reference that can lead to improvements in assisted reproductive technologies and provide evolutionary insights across species. Importantly, bovine embryos, which are more similar to human embryos than mouse embryos are, in terms of gene expression profiles and developmental timing, can serve as a great model for understanding human development, especially since human *in vivo* embryos are not available for research.

## Materials and Methods

### Collection of Bovine Gametes and Embryos

Frozen bovine sperm from a Holstein bull with proven fertility were thawed and washed using PureCeption gradient solution to remove somatic cell contaminants. After serial dilutions, three aliquots of approximately 20 sperm each were snap frozen and stored at -80°C until sequencing library preparation.

Ovarian stimulation and oocyte retrieval from Holsten cows (*n* = 10) were performed as previously described (Hayakawa et al., 2009; Jiang et al., 2014, 2018). Briefly, superovulation was achieved using five doses of intramuscular injections of FSH beginning 5 days after insertion of a Controlled Intra-vaginal Drug Release (CIDR) device. Two doses of prostaglandin F2 alpha were given along with the last two FSH treatments, followed by CIDR removal. Standing estrus (Day 0) was seen approximately 48 h post-prostaglandin injection. GnRH was then administered at estrus. Each cow was inseminated 12- and 24-h post-standing heat. Donor cows were sacrificed at 30 h and 2–4 days after estrus to collect *in vivo* matured oocytes and 2- to 16-cell embryos, respectively, by oviductal flushing. For *in vitro* matured oocytes, GV oocytes were collected as cumulus-oocyte complexes from follicles of 3–5 mm in diameter from slaughterhouse ovaries. BO-IVM medium (IVF Bioscience) was used for oocyte *in vitro* maturation. This was conducted in four-well dishes for 24 h at 38.5°C with 5% CO_2_. Oocyte and embryo stages were then evaluated under light microscopy and only Grade 1 embryos by standards of the International Embryo Technology Society were selected for further study.

All single oocytes and embryos were washed with D-PBS containing 1 mg/ml polyvinylpyrrolidone (PBS-PVP) and transferred into 50 μl droplets of 0.1% protease to remove the zona pellucida. Single oocytes and embryos were rinsed three times in PBS-PVP and confirmed to be free of contaminating cells, and then snap frozen with minimal medium and stored at -80°C until sequencing library preparation.

### Preparation of WGBS Libraries

We obtained pools of 20 sperm (*n* = 3), single germinal vesicle (GV) oocytes (*n* = 4), single *in vivo* matured oocytes (*n* = 6), single *in vitro* matured oocytes (*n* = 6) and single embryos at the 2-cell (*n* = 4), 4-cell (*n* = 5), 8-cell (*n* = 4), and 16-cell (*n* = 3) stages (Supplementary Table S1). We followed the protocol of library preparation by Smallwood et al. (2014) to prepare the single oocyte/embryo WGBS libraries. Briefly, sperm or a single oocyte/embryo were seeded into lysis buffer with 20 mg/ml of protease and 10% Trition-X 100. Genomic DNA was released after incubation at 50°C for 3 h, followed by 75°C for 30 min to inactivate the protease. Bisulfite treatment to convert unmethylated cytosines to uracils was conducted by using the MethylCode Bisulfite Conversion Kit (Thermo Fisher). The synthesis of complementary strands was repeated five times with Biotinylated random primer Bio-P5-N9 (Biotin-CTACACGACGCTCTTCCGATCTNNNNNNNNN). This allowed the maximizing of the tagged DNA strands and the generation of multiple copies of each fragment. Second-strand DNA was synthesized using another random primer, P7-N9 (AGACGTGTGCTCTTCCGATCTNNNNNNNNN). Final libraries were prepared after 12 cycles of PCR amplification using Illumina Universal PCR primers and indexed primers (NEBNext Multiplex Oligos for Illumina, New England BioLabs). Agencourt AMPure XP beads were used to purify the amplified libraries. The quality and quantity of the libraries were determined using high-sensitivity DNA chips on the Agilent Bioanalyzer, and KAPA Library Quantification Kits (KAPA Biosystems). Indexed libraries were pooled and sequenced on the Illumina HiSeq4000 platform with 150 bp paired-end reads. On average, 10× coverage of bovine genome per sample was obtained. With at least 3 replicates at each stage, our sequencing depth satisfied the requirement of Roadmap Epigenomics guidelines for WGBS that at least 30× coverage of the genome when reads from biological replicates are combined (Bernstein et al., 2010). The raw FASTQ files are available at Gene Expression Omnibus (GEO)^1^ under accession number GSE121758.

### Read Filtering and Mapping

The sequencing adapters were removed by TrimGalore-0.4.3^2^. The reads were removed if they had a quality score lower than 20, length less than 36 and a total number of 15 Ns or more. FastQC^3^ was used to assess the read quality. The first 12 bp at the 5′ end of both pairs were found to be of low quality and removed. Trimmed sequences were mapped to the bovine genome UMD3.1.1 using Bismark - v.0.18.1 (Krueger and Andrews, 2011), with parameters: –non_directional, –score_min L,0,-0.6, –un. This resulted in an average of 11.8 million reads per sample uniquely mapped (Supplementary Table S1). After mapping, we removed duplicates and non-converted reads using deduplicate_bismark and filter_non_conversion, respectively (Krueger and Andrews, 2011). An average of 4 million reads per sample remained for downstream analysis (Supplementary Table S1). This corresponds to 33.8% sequencing read usage, 13% higher than the original report by Smallwood et al. (2014).

### Quantification of Methylation Level and CpG Density

Using Bismark Methylation Extractor (Krueger and Andrews, 2011), methylation coverage for every single C was extracted and read coverage files were generated. Non-CpG methylation was also reported. When calculating the methylation level of each CpG site, the read coverage files of cytosines in CpG context were used. The DNA methylation level of each CpG site was calculated using count_methylated (“C” reads) divided by sum of count_methylated and count_unmethylated (“C” + “T” or total read counts). The numbers of CpG sites with 1×, 5×, or 10× total read counts of each stage were summarized in Supplementary Table S1. Data visualization and analysis were preformed using custom R, Java scripts and SeqMonk^4^.

To facilitate the comparison of methylation levels across samples, we applied the consecutive genomic window method to bin the bovine genome (Zhu et al., 2018). Briefly, we first filtered out CpGs that had total read counts of less than 5. Then, we bound the genome to 300-bp tiles. Only tiles that contained greater than three CpG sites were kept. Tiles from replicate samples of the same developmental stage were combined to increase coverage (Figure 2A). The number of captured 300-bp tiles in each stage is summarized in Supplementary Figure S1E. We then identified the common 300-bp tiles among all samples as commonly methylated. Uniquely methylated tiles were also obtained for each sample. DNA methylation of each sample was calculated by averaging the 300-bp tiles’ methylation. Moreover, we calculated the CpG density as described by Guo et al. (2014). First, we determined the total number of all CpG sites located within 150 bp upstream and downstream of each CpG site. Then, the CpG density of every 300-bp tile was determined as the average of all CpG sites within the 300-bp tile.

### Pairwise Comparison of Methylation Changes and Gamete-Specific DMRs

Using Bedtools (Quinlan and Hall, 2010), we identified the common 300-bp tiles between consecutive stages and between male and female gametes. We followed the method by Guo et al. (2014) to classify changing tiles as those with methylation differences greater than 40% and significantly different by Fisher’s exact test (*P*-value ≤ 0.05, FDR ≤ 0.05). The remaining tiles were defined as stable tiles. Increasing/decreasing tiles between consecutive stages were used to define DNA methylation changes. Differentially methylated regions (DMRs) were defined as common 300-bp tiles between two types of gametes/stages that had methylation levels ≥ 75% in one stage/type and ≤25% in another, and were significantly different by Fisher’s exact test (*P*-value ≤ 0.05, FDR ≤ 0.05). Hyper- and hypo-methylated tiles were those with DNA methylation levels ≥ 75% and ≤25%, respectively.

### Genomic Feature Annotation

Genomic features, including promoters (1,000 bp upstream of Transcription Start Sites; TSS), exons, introns, CpG islands (CGIs), intergenic, long interspersed nuclear elements (LINEs), short interspersed nuclear elements (SINEs), and long terminal repeats (LTRs) were downloaded from University of California, Santa Cruz (UCSC) genome browser (bovine genome UMD3.1.1).

### Gene Ontology Analysis

Gene Ontology (GO) of DMRs was performed using DAVID (Huang et al., 2009a,b). GO terms with an FDR adjust *P*-value ≤ 0.05 were deemed statistically significant.

### Gene Expression Analysis

We downloaded RNA-seq data of bovine sperm (GSE68507, Lesch et al., 2016), GV oocytes, *in vitro* matured oocytes (GSE52415, Graf et al., 2014), and *in vivo* matured oocytes and embryos (GSE59186, Jiang et al., 2014). Raw reads were trimmed by Trimmomatic (Bolger et al., 2014) and aligned to bovine reference genome assembly UMD3.1.1 using Hisat2 version 2.0.5 aligner (Pertea et al., 2016). IsoEM version 1.1.5 (Nicolae et al., 2011) was used to quantify gene expression to fragment per kilobase million (FPKM) using default parameters. Transcripts that annotated to LINEs, SINEs, and LTRs were determined by Bedtools (Quinlan and Hall, 2010). Spearman correlation coefficients between log2 transformed gene expression levels and DNA methylation levels of promoter, gene body, exon, intron, CGIs, LINEs, SINEs, and LTRs were calculated and plotted in R.

## Results and Discussion

### Profiles of the WGBS Libraries of Bovine Gametes and Embryos

Using WGBS, we analyzed a total of 35 samples of sperm, GV oocytes, *in vivo* and *in vitro* matured oocytes and cleavage stage *in vivo* embryos. The bisulfite conversion efficiency was more than 97% in each sample (Supplementary Table S1). Pearson correlations indicated higher reproducibility within stages than between stages (Supplementary Figure S1A). Captured CpGs were broadly spread across each chromosome (Supplementary Figures S1B,C). Overall, two distinct profiles of methylation were observed: (1) highly methylated sperm, and (2) lowly methylated oocytes and embryos (Supplementary Figure S1C). Consistent with findings in humans (Hong et al., 2013; Liu et al., 2016) and mice (Mechta et al., 2017), virtually no (less than 1.5%) DNA methylation was found in mitochondrial DNA (Supplementary Figures S1B,C).

In single oocytes and embryos, an average of 9 million reads uniquely mapped to the bovine genome assembly, UMD3.1.1, with an average 9.3% mapping rate (Supplementary Table S1). This is higher than the average mapping efficiency (1.4%) of mouse single oocytes subjected to the same protocol (Smallwood et al., 2014). After removing duplicated and non-bisulfite converted reads, we obtained an average of 1.8 million and 116, 655 CpG dinucleotides at 1 and 10× coverage, respectively, in embryos and oocytes (Supplementary Table S1). In the sperm samples, the average mapped reads (40 million), mapping rates (25.3%), unique reads (35.6 million) and the numbers of CpGs with 1 and 10× coverage (12 million and 608,253) were much higher than those of the single oocytes and embryos.

### Unique Features of the Methylome Dynamics in Bovine Gametes and Pre-implantation Embryos

**Figure 1:**
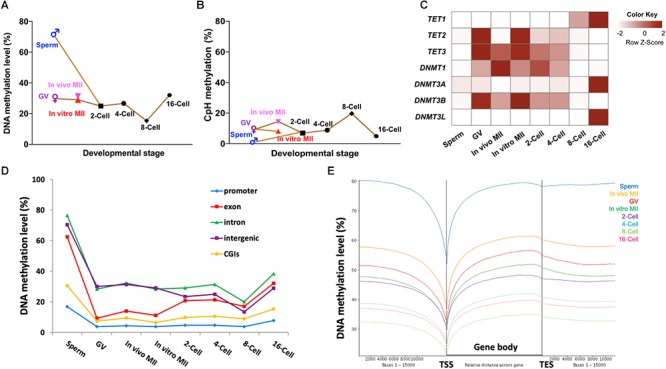
Methylome dynamics during bovine pre-implantation embryonic development. Line chart of averaged levels of CpG **(A)** and CpH **(B)** methylation across stages. Heatmap **(C)** of fragment per kilobase million (FPKM) expressions of *DNMT* and *TET* gene families in bovine early embryos. Line chart **(D)** of the average DNA methylation levels of annotated genomic features across stages. Trend plot **(E)** of averaged DNA methylation levels along the gene bodies [from transcription start sites (TSS) to transcription end sites (TES)] and 15,000 base pairs (bp) up- and down-stream of the gene body. GV, germinal vesicle oocytes; MII, matured oocytes.

A circus plot was generated to display CpG methylation levels within 300-bp tiles across all 30 bovine chromosomes (Supplementary Figure S1D). Supplementary Figure S1E summarizes the total number of 300-bp tiles in each stage. Methylation in sperm was much higher (72.5%; Figure 1A) than oocytes (29.0–31.3%) and embryos (15.3–32.1%, Figure 1A and Supplementary Figure S1D). These changes are caused by the global demethylation in the early bovine embryos. After fertilization, CpG methylation in gametes (72.5% in sperm and ∼30% in oocytes; Figure 1A) decreased rapidly at the 2-cell (25.0%; Figure 1A) and 4-cell stages (26.7%; Figure 1A). As development progressed, a further and major overall demethylation event occurred with methylation reaching the lowest point at the 8-cell stage (15.3%; Figure 1A), coinciding with the onset of major embryonic genome activation (EGA) (Misirlioglu et al., 2006; Jiang et al., 2014). A doubling of DNA methylation was seen at the 16-cell stage (32.1%; Figure 1A). The timing of this major event of *de novo* methylation was consistent with our previous finding using RRBS (Jiang et al., 2018), as well as with the results generated by immunostaining (Dean et al., 2001; Dobbs et al., 2013; O’Doherty et al., 2015).

Of note, the three types of oocytes studied had minor differences in their DNA methylation levels. *In vivo* matured oocytes (31.6%) appeared to have gained an additional 2% methylation from the GV stage (29.7%) in a relatively short time frame. During this transition, the chromatin undergoes further condensation to form chromosomes; the addition of minor methylation may have mainly occurred during oocyte growth. But this minor methylation change is consistent with the prior observation that the maturation/growth process involves addition of DNA methylation in the mouse (Kono et al., 1996). Moreover, a small but noticeable difference in methylation levels was also seen between *in vitro* (29.0%) and *in vivo* matured oocytes. This difference may suggest aberrant DNA methylation during *in vitro* maturation. *In vitro* maturation, fertilization, and culture has been linked to abnormal embryo development and gene expression (Smith et al., 2005, 2009) and large offspring syndrome (Young et al., 1998).

Interestingly, the methylation levels of non-CpG (CpH) sites showed an opposite demethylation-remethylation pattern and remained mostly at low levels (Figure 1B and Supplementary Figure S1F). For example, non-CpG methylation peaked at the 8-cell stage and was the lowest in sperm, a reverse pattern to that observed for CpG methylation. A similar pattern between CpG and non-CpG methylation was also observed in monkey embryos (Gao et al., 2017). Although non-CpG methylation has been reported to be enriched in oocytes (Tomizawa et al., 2011) and pluripotent stem cells, its functions, if any, remain poorly understood. Our results showed that non-CpG methylation peaked when high expression of pluripotency genes and EGA occurred in bovine pre-implantation embryos (Jiang et al., 2014), indicating an active regulatory role of non-CpG methylation in pluripotent gene expression as speculated earlier (Ramsahoye et al., 2000).

### Potential Mechanisms for the Methylome Dynamics

To better understand the mechanisms underpinning the DNA methylation dynamics, we analyzed the RNA-seq data of bovine gametes and embryos (Graf et al., 2014; Jiang et al., 2014; Lesch et al., 2016) for genes that encode DNA methylcytosine dioxygenases (*TET1*, *TET2*, and *TET3*; (Huang et al., 2014) and DNA methyltransferases (*DNMT1*, *DNMT3A*, *DNMT3B*, and *DNMTL*). *TET3* and *TET2* were enriched in oocytes and 2-cell embryos, and their levels started to fade away at the 4-cell stage (Figure 1C), indicating a *TET*-mediated active DNA demethylation event that continued after fertilization (Duan et al., 2019). Interestingly, expression of the other TET family member, *TET1*, was first seen at 4-cell stage and peaked at the 16-cell stage (Figure 1C), corresponding to its known function of promoting pluripotency of the inner cell mass (ICM) in blastocysts (Ito et al., 2010; Seisenberger et al., 2013). On the other hand, the expression level of transcripts for *DNMT1*, the methylation maintenance enzyme (Goyal et al., 2006), was highest in *in vivo* matured oocytes, reduced gradually after fertilization, and reached the lowest level at the 8-cell stage (Figure 1C). This may be why the overall methylation levels of matured oocytes and the first two cleavage stage embryos did not dramatically decline until the 8-cell stage since bisulfite treatment cannot distinguish between 5-methylcytosine and the product of TET activity, 5-hydroxymethylcytosine. Transcripts for the *de novo* methyltransferase *DNMT3A* (Cheng and Blumenthal, 2011), and its cooperative homolog, *DNMT3L* (Hata et al., 2002), were low until the 16-cell stage (Figure 1C), when we observed a doubling of DNA methylation levels. The third gene in the *DNMT3* family, *DNMT3B*, had an expression pattern similar to that of *DNMT1*, and may be important for *de novo* methylation at earlier stages (Liao et al., 2015). Taken together, the expression dynamics of methyltransferases is closely related to changes of the methylome in bovine oocytes and early embryos, and active demethylation by different members of the TET family may be involved throughout early embryo development as opposed to largely at the zygotic and blastocyst stage as seen in the mouse (Iqbal et al., 2011; Wossidlo et al., 2011).

### Genomic Regions of Dynamic Methylation Changes

To determine the specific genomic regions that underwent dynamic methylation changes, we analyzed the methylation levels of promoters, exons, introns, CGIs, and intergenic regions of all annotated bovine genes (Figure 1D). We found that promoters and CGIs were consistently lowly methylated across all developmental stages, even in the highly methylated sperm. This observation was also found in other species (Smith et al., 2012; Guo et al., 2014). Interestingly, exons were highly methylated in sperm (∼60%), but lowly methylated in oocytes (< 20%) and cleavage stage embryos (∼30%), suggesting that exons of the paternal genome underwent specific and deeper de-methylation than other elements of the genome. Methylation levels in exons had a minor increase at the 2-cell stage, but surged remarkably between the 8- and 16-cell stages. On the other hand, changes in introns and intergenic regions closely resembled the whole genome dynamics (Figure 1D). The patterns of methylation dynamics in promoters and CGIs, which make up less than 1% of the genome, did not follow those of the whole genome and stayed in a hypomethylated state (methylation level ≤ 25%). In addition, previous studies have also found that about 70% of promoter regions and CGIs that had high CpG densities remained predominantly unmethylated (Xie et al., 2013; Guo et al., 2014; Takahashi et al., 2017). These data suggest that the global methylation changes mainly reflect those of non-coding/regulatory regions, such as intergenic regions and introns. Regulatory regions, such as promoters and CGIs, as well as coding regions (exons) have their own specific pattern of fluctuations in DNA methylation. Subsequently, we examined DNA methylation along the gene body and 15 kb up-and downstream of all annotated bovine genes (Figure 1E). A valley in methylation levels was observed around the TSS of all genes, coinciding with the predominantly unmethylated promoters. Methylation gradually increased from TSS to transcription end site (TES) and slightly decreased after TES in all stages. This pattern was repeatedly observed in all samples regardless of their overall methylation levels (Figure 1E) and was also seen in our RRBS study (Jiang et al., 2018). This suggests that, in addition to gene expression regulation, DNA methylation may also be used as a marker for a gene’s TSS and TES boundaries (Naumann et al., 2009). The enrichment of DNA methylation of the gene body has been associated with constitutive expression of housekeeping genes (Bewick and Schmitz, 2017; Zilberman, 2017). While this may suggest that gene body methylation is involved in active transcription, the mechanism of this requires further investigation.

### Correlation Between CpG Density and Methylome Dynamics

**Figure 2:**
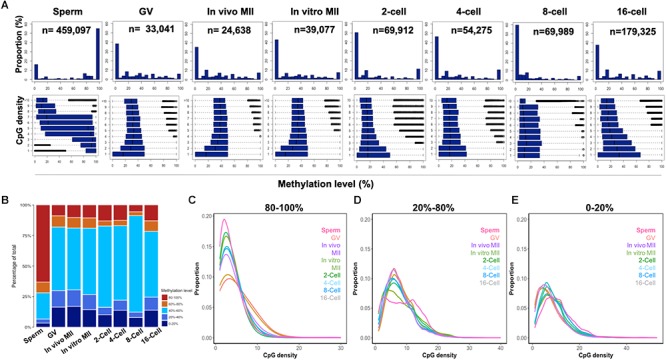
CpG density and the methylome dynamics of bovine gametes and pre-implantation embryos. Histogram **(A)** of the percentages of 300-bp tiles with different DNA methylation levels at each development stage (upper panels). Box plots of methylation levels across different CpG densities at each stage (bottom panels). Stack bar plot **(B)** of the percentages of tiles with high (80–100%), intermediate (60–80%; 40–60%, and 20–40%), and low (0–20%) methylation levels. The distribution of high **(C)**, intermediate **(D)**, and low **(E)** methylation tiles against CpG densities at each stage. GV, germinal vesicle oocytes; MII, matured oocytes.

To determine whether CpG density is correlated with DNA demethylation and remethylation patterns, we plotted the DNA methylation levels of 300-bp tiles against their CpG density in all samples (Figure 2A). Genome regions were categorized into high (80–100%), intermediate (20–40%, 40–60%, and 60–80%), and low (0–20%) methylation (Figure 2B) and their correlation to CpG density was plotted in Figure 2C–E. Sperm exhibited a strong negative correlation (*r* = -0.97) between CpG density and methylation levels: regions with low CpG density had high methylation and vice versa (Figure 2A,C–E). Surprisingly, such a negative correlation had also been reported previously in differentiated somatic cells, likely because both cell types are highly methylated (Smith et al., 2012). However, among the other samples, only the 16-cell stage embryos had some trend of negative correlation (Figure 2A). The high overall levels of methylation in the sperm was the result of their containing a high proportion (more than 60%) of highly methylated tiles, while oocytes and cleavage embryos had less than 20% of such tiles (Figure 2B). Inverse correlation between CpG density and DNA methylation levels was also seen in our RRBS study (Jiang et al., 2018) of gametes and embryos, and in embryonic stem cells (Booth et al., 2012).

### Correlation Between Dynamics of Transcriptomes and Methylomes

Using RNA-seq data of bovine sperm (Lesch et al., 2016), GV oocytes, oocytes matured *in vitro* (Graf et al., 2014) and *in vivo* as well as cleavage stage embryos (Jiang et al., 2014), we observed weak negative correlations, ranging from -0.30 in sperm to -0.18 in the *in vivo* matured oocytes, between methylation levels of promoters and the expression of the corresponding genes (Supplementary Figure S2A). There were very weak negative correlations (in the range of -0.21 to -0.11) between gene expression and methylation of gene body, exon, intron, and CGI (Supplementary Figure S2B). The correlation between methylation levels of repetitive elements and their corresponding expression, however, was weakly positive, ranging from 0.14 to 0.18 (Supplementary Figure S2B). A previous study in mouse embryos also reported similar observations (Papin et al., 2017). While LINEs, SINEs, and LTRs underwent drastic de-methylation from gametes to 8-cell stage (Supplementary Figure S2C), their overall RNA expression remained at low, but relatively constant levels (FPKM < 40), throughout the developmental stages studied (Supplementary Figure S2D), indicating the repression of repetitive element expression was possibly exerted through other mechanisms (Reik, 2007).

### Commonly and Uniquely Methylated Regions

**Figure 3:**
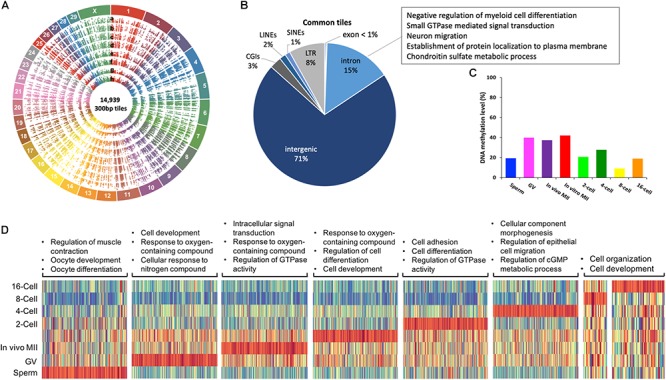
Commonly methylated regions in bovine gametes and pre-implantation embryos. Circos plot **(A)** visualization of 14,939 commonly methylated 300-bp tiles among all samples. a. sperm, b. GV, c. *in vivo* MII, d. *in vitro* MII, e. 2-cell, f. 4-cell, g. 8-cell, h. 16-cell. Pie plot **(B)** of the distribution of commonly methylated tiles in genomic regions and their associated GO term representatives. Bar plot **(C)** of averaged DNA methylation levels of commonly methylated tiles across stages. Heatmap **(D)** of enrichment of hypermethylated regions in each stage and associated GO term representatives. GV: germinal vesicle oocytes; MII: matured oocytes. Green: hypomethylation, red: hypermethylation.

A total of 14,939 300-bp tiles were found across all samples and termed commonly methylated. Their distribution along the 30 bovine chromosomes is illustrated in circos plots (Figure 3A). These tiles were characterized by low CGI density and low gene density (Figure 3B). Specifically, 86% of these tiles were located in non-coding regions (71% intergenic and 15% introns), 3% in CGIs, and 11% in repetitive regions, such as LINEs (2%), SINEs (1%), and LTRs (8%). Because commonly methylated introns were the only regions that could lead to examination of functional genes in this group of tiles, we looked at their GO terms and found that they were enriched for involvement in cell differentiation and migration, signal transduction, protein localization and metabolic processes (Figure 3B and Supplementary Table S2). Many of these were housekeeping genes suggesting the importance of consistent expression during early development. The methylation of introns of housekeeping genes found here corresponds to the earlier finding of methylation of their gene body (Zilberman, 2017). Interestingly, commonly methylated tiles exhibited a very similar pattern of methylation changes to that observed in oocytes and embryos, but not in sperm (Figure 3C). Commonly methylated tiles only had a methylation level of 19.4% in sperm, compared to the global methylation level of 72.5%, suggesting that these tiles, although mainly intergenic, possibly resisted global demethylation. This difference suggests that the sperm and oocytes/embryos are differentially methylated even in the intergenic regions.

Within commonly methylated regions, tiles that were hypermethylated (methylation level ≥ 75%) in a specific stage are represented by heatmaps for their GO categories (Figure 3D). In the sperm, these tiles were enriched in genes of muscle contraction regulation, as well as oocyte development, and differentiation (Figure 3D and Supplementary Table S3), corresponding to the need for their repression. On the other hand, more GO terms were involved in hypermethylated tiles in *in vivo* matured oocytes than GV oocytes and those matured *in vitro* (Supplementary Table S3). A common GO term among the three types of oocytes was response to oxygen-containing compound (Figure 3D and Supplementary Table S3). Oxygen stress in *in vitro* culture could generate excessive cytotoxic reactive oxygen species (ROS) and affect the viability of gametes (Park et al., 2005). The hypermethylation of these genes in oocytes is consistent with the fact the oocytes are naturally located in environment of low oxygen tension and are of better quality when they are matured in such conditions. The hypermethylation of these genes may hamper the oocytes’ ability to adapt to artificial culture environments (Waldenström et al., 2009). Additionally, the hypermethylated regions in 8- or 16-cell embryos were mostly hypermethylated in other stages (Figure 3D), suggesting these regions either resisted demethylation or regained their methylation during the *de novo* process.

We next analyzed the uniquely methylated regions in each sample (Supplementary Figure S3A). Sperm had the highest number of such tiles (276,190), followed by the 16-cell embryos (31,628), with the least in *in vivo* matured oocytes (877). Those in sperm (Supplementary Figure S3B) were enriched in intergenic (30%) and repetitive regions (31%), including 16% in SINES, 12% in LINEs and 3% in LTR, while only 1% fell in promoter regions. The GO terms of genes represented by these tiles were immune and inflammatory responses, G-protein receptor signaling pathway, and cell adhesion (Supplementary Figure S3B). Uniquely methylated regions in oocytes and embryos were also enriched in intergenic and repetitive regions (Supplementary Figure S3C) and their changes in methylation level (Supplementary Figure S3D) closely resembled those in the commonly methylated regions (Figure 3C) and the global changes in methylation. However, uniquely methylated regions in sperm (95% of the total tiles) were hypermethylated (82.3%); while, their commonly methylated region (5% of the total tiles) were hypomethylated (19.4%), indicating that the uniquely methylated regions in the sperm were likely targets of methylation erasure and re-establishment during embryonic development.

### Pairwise Methylome Comparisons at Consecutive Stages of Development

**Figure 4:**
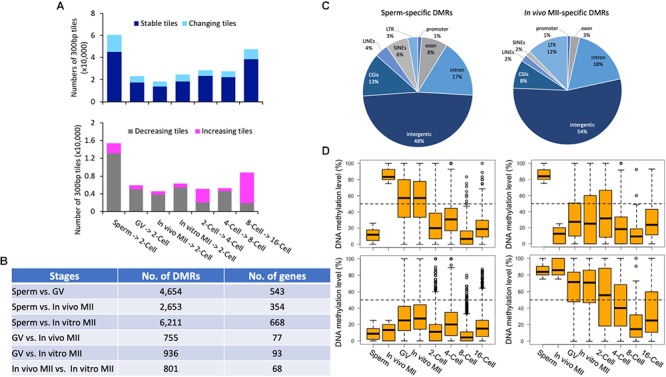
Pairwise comparisons of methylomes between consecutive development stages and DMRs in gametes. Histogram **(A)** of the numbers of stable (dark blue) and changing (sky blue) tiles between consecutive stages. Histogram of the numbers of decreasing (gray) and increasing (pink) tiles between consecutive stages. The numbers **(B)** of DMRs and corresponding genes between gametes of different types. Pie plots **(C)** of the distribution of *in vivo* MII- and sperm-specific DMRs in annotated genomic regions. Box plots **(D)** of DNA methylation levels of oocyte- (upper left) or sperm- (upper right) specific DMRs in gametes and early embryos, as well as distributions of tiles hypomethylated (≤25%; bottom left) and hypermethylated (≥75%; bottom right) tiles in both gametes in each development stage. GV, germinal vesicle oocytes; MII, matured oocytes.

Overall, the methylation of the majority of the tiles (77.6% on average of each stage) were stable (differences ≤ 40%) (Figure 4A), indicating that dynamic methylome changes occurred in a small number of regions of the genome. Of the two transitions that had the most changing tiles (Figure 4A), a large portion (84.7%) showed decreased methylation from sperm to 2-cell; while, 78.7% of tiles had increased methylation from 8- to 16-cell. A total of 951 genes were represented by the differentially methylated tiles (Supplementary Figure S3E) between the 8- and 16-cell stages. Despite the overall *de novo* methylation pattern at this transition, 256 genes were de-methylated (Supplementary Figure S3E). These genes were involved in actin cytoskeleton reorganization, negative regulation of transcription, and cell adhesion, suggesting that the embryos were preparing for the active division and differentiation required for morula and blastocyst formation. Conversely, a total of 695 genes were *de novo* methylated at the 16-cell stage and were associated with intracellular protein transport, cell migration, and DNA-template transcription (Supplementary Figure S3E and Supplementary Table S4).

### Characteristics of DMRs Between Different Types of Gametes

We identified DMRs between sperm and each oocyte type, between GV and MII oocytes, and between *in vivo* and *in vitro* matured oocytes (Supplementary Figures S4A–F). The greatest number of DMRs and corresponding annotated genes were found between sperm and *in vitro* matured oocytes (6,211), while the least were between GV and *in vivo* matured oocytes (755) (Figure 4B). Large numbers of DMRs (801) were also found between *in vivo* and *in vitro* matured oocytes. Between sperm and *in vivo* matured oocytes, 1,200 DMRs were highly methylated in *in vivo* matured oocytes (Figure 4C) and 1,453 were highly methylated in sperm (Figure 4C). These DMRs may represent parent-of-origin specific epigenetic modifications. Most of them, however, were distributed in intergenic regions. A high percentage of DMRs were LTRs (oocyte: 12%; sperm: 3%) and SINEs (oocyte: 2%; sperm: 6%). More highly methylated DMRs were found in exons (sperm: 8%; oocyte: 3%) and CGIs (sperm: 13%; oocyte: 8%) in sperm than in oocytes.

We then profiled the dynamic changes of the DMRs that were highly methylated in one type of gametes (Figure 4D, upper panel). Only DMRs that were located in the commonly methylated regions across all samples were included for downstream analysis. DMRs among different types of oocytes were mostly intermediately methylated (25–75%). Interestingly, the majority of the DMRs that were highly methylated in *in vivo* matured oocytes had intermediate methylation in both GV oocytes and *in vitro* matured oocytes, indicating that the oocytes gained additional methylation during the maturation process and the *in vitro* environment was not able to ensure proper DNA methylation in these regions. A previous study also demonstrated that suboptimal *in vitro* culture altered the DNA methylation landscape in bovine embryos (Salilew-Wondim et al., 2015). Another potential contributor to this difference is the size of the follicles from which the oocytes were derived (O’Doherty et al., 2012). *In vivo* matured oocytes ovulated from fully grown follicles, while the GV and *in vitro* matured oocytes were aspirated from follicles 3–5 mm in diameter. During the antral phase of follicle growth, the oocyte also changes in size, albeit minor, and likely methylation as well. More changes in methylation were found in oocytes that were matured *in vitro* (Kerjean et al., 2003; Borghol et al., 2006; Song et al., 2009). Furthermore, changes in DMRs that were hypermethylated in *in vivo* matured oocytes (Figure 4D upper left panel), resembled the global methylome dynamics during pre-implantation development. In contrast, DMRs specifically hypermethylated in sperm (Figure 4D upper right panel) had relatively high methylation at the 2-cell stage compared to those that were only hypermethylated in the oocytes (Figure 4D upper left panel).

Additionally, we identified 1,063 and 9,310 tiles that were either hypermethylated or hypomethylated in both sperm and *in vivo* matured oocytes (Figure 4D bottom panels). The hypomethylated tiles were not further demethylated in pre-implantation embryos, but increased their methylation level at the 16-cell stage (Figure 4D bottom left panel). However, tiles that were hypermethylated in both gametes, and localized in mostly intergenic regions, became largely hypomethylated during subsequent development and reached the methylation level nadir at the 8-cell stage (Figure 4D bottom right panel).

The annotated genes encompassing all DMRs from the six comparisons are summarized in Supplementary Table S5. Interestingly, only one common GO term, cell adhesion, was found in DMRs that were hypermethylated in sperm while hypomethylated in the three types of oocytes (Supplementary Figures S4A–C). A previous methylome study (Perrier et al., 2018) compared bull sperm to somatic tissues, and showed methylated regions specific to sperm were also involved in cell adhesion, along with migration and fertilization, which are essential for sperm viability and function. For DMRs between *in vivo* and *in vitro* matured oocytes, genes involved in positive regulation of endosome, cellular component organization, and cytoplasmic transport were hypermethylated in *in vivo* matured oocytes, while those related to urogenital and reproductive system development, and cell development were hypermethylated in *in vitro* matured oocytes. While most of these GO terms do not seem to be related to functions of gametes, the discrepancies may set the foundation for the abnormal fetal development in embryos produced *in vitro*. We have shown previously that oocytes matured and fertilized *in vivo* but cultured *in vitro* had a blastocyst rate of 75%; while, embryos from *in vitro* matured oocytes had a 37% blastocyst rate when cultured *in vitro* under the same conditions (Smith et al., 2009). Rizos et al. (2002) also observed the crucial role of oocyte maturation conditions in blastocyst yield. These data demonstrate the importance of proper oocyte maturation.

### Methylation of the X Chromosome and Imprinted Genes

**Figure 5:**
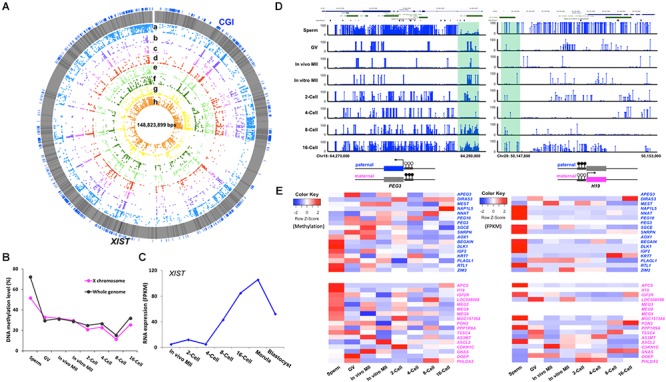
Methylation of the X chromosome and imprinted genes in bovine gametes and pre-implantation embryos. Circos plot **(A)** visualization of the methylation dynamics of genomic region of the X chromosome. All genes are in gray lines, Xist gene is in black line. CGIs are in blue lines. (a) sperm, (b) GV, (c) *in vivo* MII, (d) *in vitro* MII, (e) 2-cell, (f) 4-cell, (g) 8-cell, (h) 16-cell. Line plot **(B)** showing the DNA methylation dynamics of the X chromosome followed the global pattern of methylation changes. Line chart **(C)** of fragment per kilobase million (FPKM) expressions levels of *XIST* (TPM) in bovine early embryos. Visualization of **(D)** imprinted control regions (ICR) of *PEG3* and *H19*. Heatmap of **(E)** of the methylation and expression levels (TPM) of 34 imprinted genes in pre-implantation embryos. GV, germinal vesicle oocytes; MII, matured oocytes. Blue text: paternally expressed genes, pink text: maternally expressed genes, Color key for heatmap: blue, hypomethylation and low expression, red, hypermethylation and high expression.

In the mouse, the sperm carries an inactive X chromosome which is quickly reactivated after fertilization (Goto and Monk, 1998). We found that the bovine gametes also had differentially methylated X chromosomes with the X in sperm much more methylated than that in matured oocytes. After fertilization, the overall DNA demethylation pattern of the paternal X chromosome closely resembled that of the whole genome (Figure 5A,B), suggesting reactivation of the paternal X chromosome. This is consistent with the observation that expression of the X-linked *MAOA* gene was detected from both parental X chromosomes until the morula stage when XCI was first observed (Ferreira et al., 2010). Interestingly, the overall methylation level of the paternal X chromosome was about 10% lower than that of the entire sperm genome (Figure 5B), probably because it contained more X-linked hypomethylated tiles than the whole genome (Supplementary Figure S1D). This observation was also seen in our bovine RRBS data (Jiang et al., 2018) and a WGBS study of monkey gametes and embryos (Gao et al., 2017). During spermatogenesis, partially synapsed X and Y chromosomes undergo meiotic sex chromosome inactivation (MSCI) and form the distinct chromatin domain, namely, XY- or sex- body (Cloutier and Turner, 2010; Ichijima et al., 2012). This results in the nearly complete inactivation of transcription from the sex chromosomes in the spermatocytes, which persists during the two rounds of meiotic division (Fallahi et al., 2010). In spermatids, 87% of X-linked genes remain repressed while autosomal genes are largely active (Namekawa et al., 2006). Our data and that of Gao et al. (2017) suggests that even though the gene expression from the sperm X chromosome is more suppressed than from the autosomes, methylation of the sperm X chromosome was lower and may not be the major mechanism for transcription inhibition. The more important mechanism may be the formation of the heterochromatic sex body, which acts as a barrier by preventing access of the transcriptional machinery.

The expression of the X-linked *XIST* gene is essential for initiation of XCI in mammalian embryos (Kalantry et al., 2009). In males, *XIST* is expressed in the testes and is speculated to coat the X chromosome and form sex body during male MSCI (Turner, 2007). Using expression data from Jiang et al. (2014) and Lesch et al. (2016), we found that the *XIST* transcript was absent in sperm and its transcription was not initiated until the 2-cell stage (Figure 5C); this is consistent with data by De La Fuente et al. (1999) in early bovine embryos. The major increase in *XIST* expression was observed between the 4- to 8- cell stage, when EGA occurs, and it peaked at the morula stage (Figure 5C); this is likely because XCI will be established soon, which was first reported by Xue et al. (2002) in the bovine.

Genomic imprinting is a phenomenon of parent-of-origin specific gene expression (Rivera and Bennett, 2010) and is regulated by differential epigenetic marks on gametes (Zaitoun et al., 2010). Unlike the whole genome, which undergoes a drastic reprogramming after fertilization, imprinted genes retain their germline DMRs (Sanz et al., 2010; Stewart et al., 2016). To date, 53 imprinted genes have been identified in the bovine (Chen et al., 2016), of which 34 are annotated in the current genome and were analyzed in this study.

Interestingly, we observed two distinct patterns of gamete-specific methylation for bovine imprinted genes. The first included 20 genes, 9 paternally and 11 maternally expressed, whose average methylation along the gene body was negatively correlated with their reported allelic expression, as expected. For example, the paternally expressed gene, *SGCE*, was hypomethylated in sperm and hypermethylated in *in vivo* matured oocytes; while, the maternally expressed gene *IGF2R* was hypomethylated in oocytes and hypermethylated in sperm. In the second pattern, which included 14 genes, 8 paternally, and 6 maternally expressed, a positive correlation of methylation and the known allelic expression pattern was observed (Figure 5E). For instance, the paternally expressed genes, *BEGAIN*, *IGF2*, and *RTL1*, were hypermethylated in the gene bodies in sperm and hypomethylated in oocytes; while, the maternally expressed *OOEP* and *PHLDA2* were hypermethylated in their gene bodies in the oocytes. Our RRBS data from bovine embryos (Jiang et al., 2018) also documented similar findings. The disagreement between methylation and expression patterns could be due to the involvement of other epigenetic mechanisms (Inoue et al., 2017), as well as the fact that the imprinting control region may fall into other regions including those that are intergenic. Overall, the expression heatmap (Figure 5E) showed that the majority of paternally expressed genes had high expression levels in sperm, while a significant number of the maternally expressed genes had high expression in the GV oocytes.

We then characterized genes for which the imprinting control regions (ICR) (Pervjakova et al., 2016) are known regulatory germline DMRs. In the bovine, these are very few. The ICRs for imprinted genes *PEG3* (Kim et al., 2007) and *H19* (Robbins et al., 2012) (Figure 5D) are located at the first exon (Kim et al., 2007) and 3 kb upstream of the *H19* promoter (Hansmann et al., 2011; O’Doherty et al., 2015), respectively. In bovine gametes and early embryos, the methylation of these ICRs in gametes corresponded to their parent-of-origin specific expression. For example, the ICR of the paternally expressed *PEG3* was hypomethylated in sperm and hypermethylated in oocytes, and the methylation was maintained at around 50% up to the 16-cell stage (Figure 5D), as expected.

## Conclusion

Our data delineated the complex methylome reprogramming of bovine gametes and early embryos. The major wave of genome-wide DNA demethylation was completed at the 8-cell stage when *de novo* methylation became prominent. Sperm and oocytes were differentially methylated in numerous regions, and DMRs were also identified between *in vivo* and *in vitro* matured oocytes. The WGBS results paralleled our previous published RRBS data (Jiang et al., 2018), and provided further insights at single CpG resolution, which allowed us to characterize DNA methylation of the X chromosome and known imprinted genes.

## Data Availability

The datasets generated for this study can be found in Gene Expression Omnibus, GEO accession GSE121758: https://www.ncbi.nlm.nih.gov/geo/query/acc.cgi?acc=GSE121758.

## Ethics Statement

The animal protocol was approved by the Animal Care and Use Committee of Xinjiang Academy of Animal Science (Research license 200815).

## Author Contributions

XT, ZJ, and JD conceived the study. HD, XZ, and JC collected samples. ZJ performed all the experiments. JD performed data analysis with assistance from FA, IM, ZJ, SM, and XT. JD and XT interpreted the data and wrote the manuscript. ZJ and SM revised the manuscript. All authors read and approved the final manuscript.

## Conflict of Interest Statement

The authors declare that the research was conducted in the absence of any commercial or financial relationships that could be construed as a potential conflict of interest.

## Footnotes

1 www.ncbi.nlm.nih.gov/geo

2 https://www.bioinformatics.babraham.ac.uk/projects/trim_galore/

3 http://www.bioinformatics.babraham.ac.uk/projects/fastqc/

4 https://www.bioinformatics.babraham.ac.uk/projects/seqmonk/

## References

[B1] Beaujean N. Hartshorne G. Cavilla J. Taylor J. Gardner J. Wilmut I. (2004). Non-conservation of mammalian preimplantation methylation dynamics. *Curr. Biol.* 14 R266–R267. 10.1016/j.cub.2004.03.019 15062117

[B2] Bernstein B. E. Stamatoyannopoulos J. A. Costello J. F. Ren B. Milosavljevic A. Meissner A. (2010). The NIH roadmap epigenomics mapping consortium. *Nat. Biotechnol.* 28 1045–1048. 10.1038/nbt1010-1045 20944595 PMC3607281

[B3] Bewick A. J. Schmitz R. J. (2017). Gene body DNA methylation in plants. *Curr. Opin. Plant Biol.* 36 103–110. 10.1016/j.pbi.2016.12.007 28258985 PMC5413422

[B4] Bolger A. M. Lohse M. Usadel B. (2014). Trimmomatic: a flexible trimmer for Illumina sequence data. *Bioinformatics* 30 2114–2120. 10.1093/bioinformatics/btu170 24695404 PMC4103590

[B5] Booth M. J. Branco M. R. Ficz G. Oxley D. Krueger F. Reik W. (2012). Quantitative sequencing of 5-methylcytosine and 5-hydroxymethylcytosine at single-base resolution. *Science* 336 934–937. 10.1126/science.1220671 22539555

[B6] Borghol N. Lornage J. Blachère T. Sophie Garret A. Lefèvre A. (2006). Epigenetic status of the H19 locus in human oocytes following in vitro maturation. *Genomics* 87 417–426. 10.1016/j.ygeno.2005.10.008 16378710

[B7] Chen Z. Hagen D. E. Wang J. Elsik C. G. Ji T. Siqueira L. G. (2016). Global assessment of imprinted gene expression in the bovine conceptus by next generation sequencing. *Epigenetics* 11 501–516. 10.1080/15592294.2016.1184805 27245094 PMC4939914

[B8] Cheng X. Blumenthal R. M. (2011). “Chapter 1 - Introduction—Epiphanies in Epigenetics,” in *Progress in Molecular Biology and Translational Science Modifications of Nuclear DNA and its Regulatory Proteins*, eds Cheng X. Blumenthal R. M. (Cambridge, MA: Academic Press), 1–21. 10.1016/B978-0-12-387685-0.00001-9 PMC4031656 21507348

[B9] Cloutier J. M. Turner J. M. A. (2010). Meiotic sex chromosome inactivation. *Curr. Biol.* 20 R962–R963. 10.1016/j.cub.2010.09.041 21093783

[B10] De La Fuente R. Hahnel A. Basrur P. K. King W. A. (1999). X inactive-specific transcript (Xist) expression and X chromosome inactivation in the preattachment bovine embryo. *Biol. Reprod.* 60 769–775. 10026129 10.1095/biolreprod60.3.769

[B11] Dean W. Santos F. Stojkovic M. Zakhartchenko V. Walter J. Wolf E. (2001). Conservation of methylation reprogramming in mammalian development: aberrant reprogramming in cloned embryos. *Proc. Natl. Acad. Sci. U.S.A.* 98 13734–13738. 10.1073/pnas.241522698 11717434 PMC61110

[B12] Dobbs K. B. Rodriguez M. Sudano M. J. Ortega M. S. Hansen P. J. (2013). Dynamics of DNA methylation during early development of the preimplantation bovine embryo. *PLoS One* 8:e66230. 10.1371/journal.pone.0066230 23799080 PMC3683128

[B13] Doherty R. Couldrey C. (2014). Exploring genome wide bisulfite sequencing for DNA methylation analysis in livestock: a technical assessment. *Front. Genet.* 5:126. 10.3389/fgene.2014.00126 24860595 PMC4026711

[B14] Doi A. Park I.-H. Wen B. Murakami P. Aryee M. J. Irizarry R. (2009). Differential methylation of tissue- and cancer-specific CpG island shores distinguishes human induced pluripotent stem cells, embryonic stem cells and fibroblasts. *Nat. Genet.* 41 1350–1353. 10.1038/ng.471 19881528 PMC2958040

[B15] Duan J. Zhu L. Dong H. Zheng X. Jiang Z. Chen J. (2019). Analysis of mRNA abundance for histone variants, histone- and DNA-modifiers in bovine in vivo and in vitro oocytes and embryos. *Sci. Rep.* 9:1217. 10.1038/s41598-018-38083-4 30718778 PMC6362035

[B16] Fallahi M. Getun I. V. Wu Z. K. Bois P. R. J. (2010). A global expression switch marks pachytene initiation during mouse male meiosis. *Genes* 1 469–483. 10.3390/genes1030469 24710097 PMC3966219

[B17] Ferreira A. R. Machado G. M. Diesel T. O. Carvalho J. O. Rumpf R. Melo E. O. (2010). Allele-specific expression of the MAOA gene and X chromosome inactivation in in vitro produced bovine embryos. *Mol. Reprod. Dev.* 77 615–621. 10.1002/mrd.21192 20578062

[B18] Gao F. Niu Y. Sun Y. E. Lu H. Chen Y. Li S. (2017). De novo DNA methylation during monkey pre-implantation embryogenesis. *Cell Res.* 27 526–539. 10.1038/cr.2017.25 28233770 PMC5385613

[B19] Goto T. Monk M. (1998). Regulation of X-chromosome inactivation in development in mice and humans. *Microbiol. Mol. Biol. Rev.* 62 362–378. 9618446 10.1128/mmbr.62.2.362-378.1998 PMC98919

[B20] Goyal R. Reinhardt R. Jeltsch A. (2006). Accuracy of DNA methylation pattern preservation by the Dnmt1 methyltransferase. *Nucleic Acids Res.* 34 1182–1188. 10.1093/nar/gkl002 16500889 PMC1383621

[B21] Graf A. Krebs S. Zakhartchenko V. Schwalb B. Blum H. Wolf E. (2014). Fine mapping of genome activation in bovine embryos by RNA sequencing. *Proc. Natl. Acad. Sci. U.S.A.* 111 4139–4144. 10.1073/pnas.1321569111 24591639 PMC3964062

[B22] Guo H. Zhu P. Yan L. Li R. Hu B. Lian Y. (2014). The DNA methylation landscape of human early embryos. *Nature* 511 606–610. 10.1038/nature13544 25079557

[B23] Hackett J. A. Surani M. A. (2013). DNA methylation dynamics during the mammalian life cycle. *Philos. Trans. R. Soc. Lond. B Biol. Sci.* 368:20110328. 10.1098/rstb.2011.0328 23166392 PMC3539357

[B24] Hansmann T. Heinzmann J. Wrenzycki C. Zechner U. Niemann H. Haaf T. (2011). Characterization of differentially methylated regions in 3 bovine imprinted genes: a model for studying human germ-cell and embryo development. *Cytogenet. Genome Res.* 132 239–247. 10.1159/000322627 21160170

[B25] Hata K. Okano M. Lei H. Li E. (2002). Dnmt3L cooperates with the Dnmt3 family of de novo DNA methyltransferases to establish maternal imprints in mice. *Development* 129 1983–1993. 11934864 10.1242/dev.129.8.1983

[B26] Hayakawa H. Hirai T. Takimoto A. Ideta A. Aoyagi Y. (2009). Superovulation and embryo transfer in Holstein cattle using sexed sperm. *Theriogenology* 71 68–73. 10.1016/j.theriogenology.2008.09.016 18951623

[B27] Hong E. E. Okitsu C. Y. Smith A. D. Hsieh C.-L. (2013). Regionally specific and genome-wide analyses conclusively demonstrate the absence of CpG methylation in human mitochondrial DNA. *Mol. Cell. Biol.* 33 2683–2690. 10.1128/MCB.00220-13 23671186 PMC3700126

[B28] Huang D. W. Sherman B. T. Lempicki R. A. (2009a). Bioinformatics enrichment tools: paths toward the comprehensive functional analysis of large gene lists. *Nucleic Acids Res.* 37 1–13. 10.1093/nar/gkn923 19033363 PMC2615629

[B29] Huang D. W. Sherman B. T. Lempicki R. A. (2009b). Systematic and integrative analysis of large gene lists using DAVID bioinformatics resources. *Nat. Protoc.* 4 44–57. 10.1038/nprot.2008.211 19131956

[B30] Huang Y. Chavez L. Chang X. Wang X. Pastor W. A. Kang J. (2014). Distinct roles of the methylcytosine oxidases Tet1 and Tet2 in mouse embryonic stem cells. *Proc. Natl. Acad. Sci. U.S.A.* 111 1361–1366. 10.1073/pnas.1322921111 24474761 PMC3910590

[B31] Ichijima Y. Sin H.-S. Namekawa S. H. (2012). Sex chromosome inactivation in germ cells: emerging roles of DNA damage response pathways. *Cell Mol. Life Sci.* 69 2559–2572. 10.1007/s00018-012-0941-5 22382926 PMC3744831

[B32] Inoue A. Jiang L. Lu F. Suzuki T. Zhang Y. (2017). Maternal H3K27me3 controls DNA methylation-independent imprinting. *Nature* 547 419–424. 10.1038/nature23262 28723896 PMC9674007

[B33] Iqbal K. Jin S.-G. Pfeifer G. P. Szabó P. E. (2011). Reprogramming of the paternal genome upon fertilization involves genome-wide oxidation of 5-methylcytosine. *Proc. Natl. Acad. Sci. U.S.A.* 108 3642–3647. 10.1073/pnas.1014033108 21321204 PMC3048122

[B34] Ito S. D’Alessio A. C. Taranova O. V. Hong K. Sowers L. C. Zhang Y. (2010). Role of Tet proteins in 5mC to 5hmC conversion, ES-cell self-renewal and inner cell mass specification. *Nature* 466 1129–1133. 10.1038/nature09303 20639862 PMC3491567

[B35] Jiang Z. Lin J. Dong H. Zheng X. Marjani S. L. Duan J. (2018). DNA methylomes of bovine gametes and in vivo produced preimplantation embryos. *Biol. Reprod.* 99 949–959. 10.1093/biolre/ioy138 29912291 PMC6297316

[B36] Jiang Z. Sun J. Dong H. Luo O. Zheng X. Obergfell C. (2014). Transcriptional profiles of bovine in vivo pre-implantation development. *BMC Genomics* 15:756. 10.1186/1471-2164-15-756 25185836 PMC4162962

[B37] Kalantry S. Purushothaman S. Bowen R. B. Starmer J. Magnuson T. (2009). Evidence of *Xist* RNA-independent initiation of mouse imprinted X-chromosome inactivation. *Nature* 460 647–651. 10.1038/nature08161 19571810 PMC2754729

[B38] Kerjean A. Couvert P. Heams T. Chalas C. Poirier K. Chelly J. (2003). *In vitro* follicular growth affects oocyte imprinting establishment in mice. *Eur. J. Hum. Genet.* 11 493–496. 10.1038/sj.ejhg.5200990 12825069

[B39] Kim J. Bergmann A. Choo J. H. Stubbs L. (2007). Genomic organization and imprinting of the Peg3 domain in bovine. *Genomics* 90 85–92. 10.1016/j.ygeno.2007.03.012 17509818

[B40] Kono T. Obata Y. Yoshimzu T. Nakahara T. Carroll J. (1996). Epigenetic modifications during oocyte growth correlates with extended parthenogenetic development in the mouse. *Nat. Genet.* 13 91–94. 10.1038/ng0596-91 8673112

[B41] Krueger F. Andrews S. R. (2011). Bismark: a flexible aligner and methylation caller for Bisulfite-Seq applications. *Bioinformatics* 27 1571–1572. 10.1093/bioinformatics/btr167 21493656 PMC3102221

[B42] Lesch B. J. Silber S. J. McCarrey J. R. Page D. C. (2016). Parallel evolution of male germline epigenetic poising and somatic development in animals. *Nat. Genet.* 48 888–894. 10.1038/ng.3591 27294618

[B43] Liao J. Karnik R. Gu H. Ziller M. J. Clement K. Tsankov A. M. (2015). Targeted disruption of DNMT1, DNMT3A and DNMT3B in human embryonic stem cells. *Nat. Genet.* 47 469–478. 10.1038/ng.3258 25822089 PMC4414868

[B44] Liu B. Du Q. Chen L. Fu G. Li S. Fu L. (2016). CpG methylation patterns of human mitochondrial DNA. *Sci. Rep.* 6:23421. 10.1038/srep23421 26996456 PMC4800444

[B45] Mechta M. Ingerslev L. R. Fabre O. Picard M. Barrès R. (2017). Evidence suggesting absence of mitochondrial DNA methylation. *Front. Genet.* 8:166 10.3389/fgene.2017.00166 PMC5671948 29163634

[B46] Misirlioglu M. Page G. P. Sagirkaya H. Kaya A. Parrish J. J. First N. L. (2006). Dynamics of global transcriptome in bovine matured oocytes and preimplantation embryos. *Proc. Natl. Acad. Sci. U.S.A.* 103 18905–18910. 10.1073/pnas.0608247103 17142320 PMC1748150

[B47] Namekawa S. H. Park P. J. Zhang L.-F. Shima J. E. McCarrey J. R. Griswold M. D. (2006). Postmeiotic sex chromatin in the male germline of mice. *Curr. Biol.* 16 660–667. 10.1016/j.cub.2006.01.066 16581510

[B48] Naumann A. Hochstein N. Weber S. Fanning E. Doerfler W. (2009). A distinct DNA-methylation boundary in the 5′- upstream sequence of the FMR1 promoter binds nuclear proteins and is lost in fragile X syndrome. *Am. J. Hum. Genet.* 85 606–616. 10.1016/j.ajhg.2009.09.018 19853235 PMC2775827

[B49] Nicolae M. Mangul S. Mãndoiu I. I. Zelikovsky A. (2011). Estimation of alternative splicing isoform frequencies from RNA-Seq data. *Algorithms Mol. Biol.* 6:9. 10.1186/1748-7188-6-9 21504602 PMC3107792

[B50] O’Doherty A. M. Magee D. A. O’Shea L. C. Forde N. Beltman M. E. Mamo S. (2015). DNA methylation dynamics at imprinted genes during bovine pre-implantation embryo development. *BMC Dev. Biol.* 15:13. 10.1186/s12861-015-0060-2 25881176 PMC4363183

[B51] O’Doherty A. M. McGettigan P. Irwin R. E. Magee D. A. Gagne D. Fournier E. (2018). Intragenic sequences in the trophectoderm harbour the greatest proportion of methylation errors in day 17 bovine conceptuses generated using assisted reproductive technologies. *BMC Genomics* 19:438. 10.1186/s12864-018-4818-3 29866048 PMC5987443

[B52] O’Doherty A. M. O’Shea L. C. Fair T. (2012). Bovine DNA methylation imprints are established in an oocyte size-specific manner, which are coordinated with the expression of the DNMT3 family proteins. *Biol. Reprod.* 86:67. 10.1095/biolreprod.111.094946 22088914

[B53] Papin C. Ibrahim A. Gras S. L. Velt A. Stoll I. Jost B. (2017). Combinatorial DNA methylation codes at repetitive elements. *Genome Res.* 27 934–946. 10.1101/gr.213983.116 28348165 PMC5453327

[B54] Park J. I. Hong J. Y. Yong H. Y. Hwang W. S. Lim J. M. Lee E. S. (2005). High oxygen tension during in vitro oocyte maturation improves in vitro development of porcine oocytes after fertilization. *Anim. Reprod. Sci.* 87 133–141. 10.1016/j.anireprosci.2004.11.002 15885446

[B55] Park J. S. Jeong Y. S. Shin S. T. Lee K. K. Kang Y. K. (2007). Dynamic DNA methylation reprogramming: active demethylation and immediate remethylation in the male pronucleus of bovine zygotes. *Dev. Dyn.* 236 2523–2533. 10.1002/dvdy.21278 17676637

[B56] Perrier J.-P. Sellem E. Prézelin A. Gasselin M. Jouneau L. Piumi F. (2018). A multi-scale analysis of bull sperm methylome revealed both species peculiarities and conserved tissue-specific features. *BMC Genomics* 19:404. 10.1186/s12864-018-4764-0 29843609 PMC5975405

[B57] Pertea M. Kim D. Pertea G. M. Leek J. T. Salzberg S. L. (2016). Transcript-level expression analysis of RNA-seq experiments with HISAT, StringTie and Ballgown. *Nat. Protoc.* 11 1650–1667. 10.1038/nprot.2016.095 27560171 PMC5032908

[B58] Pervjakova N. Kasela S. Morris A. P. Kals M. Metspalu A. Lindgren C. M. (2016). Imprinted genes and imprinting control regions show predominant intermediate methylation in adult somatic tissues. *Epigenomics* 8 789–799. 10.2217/epi.16.8 27004446 PMC5066126

[B59] Quinlan A. R. Hall I. M. (2010). BEDTools: a flexible suite of utilities for comparing genomic features. *Bioinformatics* 26 841–842. 10.1093/bioinformatics/btq033 20110278 PMC2832824

[B60] Ramsahoye B. H. Biniszkiewicz D. Lyko F. Clark V. Bird A. P. Jaenisch R. (2000). Non-CpG methylation is prevalent in embryonic stem cells and may be mediated by DNA methyltransferase 3a. *Proc. Natl. Acad. Sci. U.S.A.* 97 5237–5242. 10805783 10.1073/pnas.97.10.5237 PMC25812

[B61] Reik W. (2007). Stability and flexibility of epigenetic gene regulation in mammalian development. *Nature* 447 425–432. 10.1038/nature05918 17522676

[B62] Rivera R. M. Bennett L. B. (2010). Epigenetics in humans: an overview: current Opinion in Endocrinology. *Diabetes Obes.* 17 493–499. 10.1097/MED.0b013e3283404f4b 20962634

[B63] Rizos D. Ward F. Duffy P. Boland M. P. Lonergan P. (2002). Consequences of bovine oocyte maturation, fertilization or early embryo development in vitro versus in vivo: implications for blastocyst yield and blastocyst quality. *Mol. Reprod. Dev.* 61 234–248. 10.1002/mrd.1153 11803560

[B64] Robbins K. M. Chen Z. Wells K. D. Rivera R. M. (2012). Expression of KCNQ1OT1, CDKN1C, H19, and PLAGL1 and the methylation patterns at the KvDMR1 and H19/IGF2 imprinting control regions is conserved between human and bovine. *J. Biomed. Sci.* 19:95. 10.1186/1423-0127-19-95 23153226 PMC3533950

[B65] Saadeh H. Schulz R. (2014). Protection of CpG islands against de novo DNA methylation during oogenesis is associated with the recognition site of E2f1 and E2f2. *Epigenetics Chromatin* 7:26. 10.1186/1756-8935-7-26 25478011 PMC4255709

[B66] Salilew-Wondim D. Fournier E. Hoelker M. Saeed-Zidane M. Tholen E. Looft C. (2015). Genome-wide DNA methylation patterns of bovine blastocysts developed in vivo from embryos completed different stages of development *In Vitro*. *PLoS One* 10:e0140467. 10.1371/journal.pone.0140467 26536655 PMC4633222

[B67] Sanz L. A. Kota S. K. Feil R. (2010). Genome-wide DNA demethylation in mammals. *Genome Biol.* 11:110. 10.1186/gb-2010-11-3-110 20236475 PMC2864560

[B68] Seisenberger S. Andrews S. Krueger F. Arand J. Walter J. Santos F. (2012). The dynamics of genome-wide DNA methylation reprogramming in mouse primordial germ cells. *Mol. Cell* 48 849–862. 10.1016/j.molcel.2012.11.001 23219530 PMC3533687

[B69] Seisenberger S. Peat J. R. Hore T. A. Santos F. Dean W. Reik W. (2013). Reprogramming DNA methylation in the mammalian life cycle: building and breaking epigenetic barriers. *Philos. Trans. R. Soc. B* 368:20110330. 10.1098/rstb.2011.0330 23166394 PMC3539359

[B70] Smallwood S. A. Lee H. J. Angermueller C. Krueger F. Saadeh H. Peat J. (2014). Single-cell genome-wide bisulfite sequencing for assessing epigenetic heterogeneity. *Nat. Methods* 11 817–820. 10.1038/nmeth.3035 25042786 PMC4117646

[B71] Smith S. L. Everts R. E. Sung L.-Y. Du F. Page R. L. Henderson B. (2009). Gene expression profiling of single bovine embryos uncovers significant effects of in vitro maturation, fertilization and culture. *Mol. Reprod. Dev.* 76 38–47. 10.1002/mrd.20927 18449896

[B72] Smith S. L. Everts R. E. Tian X. C. Du F. Sung L.-Y. Rodriguez-Zas S. L., (2005). Global gene expression profiles reveal significant nuclear reprogramming by the blastocyst stage after cloning. *Proc. Natl. Acad. Sci. U.S.A.* 102:17582. 10.1073/pnas.0508952102 16314565 PMC1308920

[B73] Smith Z. D. Chan M. M. Mikkelsen T. S. Gu H. Gnirke A. Regev A. (2012). A unique regulatory phase of DNA methylation in the early mammalian embryo. *Nature* 484 339–344. 10.1038/nature10960 22456710 PMC3331945

[B74] Song Z. Min L. Pan Q. Shi Q. Shen W. (2009). Maternal imprinting during mouse oocyte growth in vivo and in vitro. *Biochem. Biophys. Res. Commun.* 387 800–805. 10.1016/j.bbrc.2009.07.131 19646963

[B75] Stewart K. R. Veselovska L. Kelsey G. (2016). Establishment and functions of DNA methylation in the germline. *Epigenomics* 8 1399–1413. 10.2217/epi-2016-0056 27659720 PMC5066131

[B76] Takahashi Y. Wu J. Suzuki K. Martinez-Redondo P. Li M. Liao H.-K. (2017). Integration of CpG-free DNA induces de novo methylation of CpG islands in pluripotent stem cells. *Science* 356 503–508. 10.1126/science.aag3260 28473583 PMC5654639

[B77] Tomizawa S. Kobayashi H. Watanabe T. Andrews S. Hata K. Kelsey G. (2011). Dynamic stage-specific changes in imprinted differentially methylated regions during early mammalian development and prevalence of non-CpG methylation in oocytes. *Development* 138 811–820. 10.1242/dev.061416 21247965 PMC3035086

[B78] Turner J. M. A. (2007). Meiotic sex chromosome inactivation. *Development* 134 1823–1831. 10.1242/dev.000018 17329371

[B79] Waldenström U. Engström A.-B. Hellberg D. Nilsson S. (2009). Low-oxygen compared with high-oxygen atmosphere in blastocyst culture, a prospective randomized study. *Fertil. Steril.* 91 2461–2465. 10.1016/j.fertnstert.2008.03.051 18554591

[B80] Woolliams J. A. (1996). Economic aspects of animal breeding. *Livestock Prod. Sci.* 2:155.

[B81] Wossidlo M. Nakamura T. Lepikhov K. Marques C. J. Zakhartchenko V. Boiani M. (2011). 5-Hydroxymethylcytosine in the mammalian zygote is linked with epigenetic reprogramming. *Nat. Commun.* 2:241. 10.1038/ncomms1240 21407207

[B82] Xie W. Schultz M. D. Lister R. Hou Z. Rajagopal N. Ray P. (2013). Epigenomic analysis of multilineage differentiation of human embryonic stem cells. *Cell* 153 1134–1148. 10.1016/j.cell.2013.04.022 23664764 PMC3786220

[B83] Xue F. Tian X. C. Du F. Kubota C. Taneja M. Dinnyes A. (2002). Aberrant patterns of X chromosome inactivation in bovine clones. *Nat. Genet.* 31 216–220. 10.1038/ng900 12032569

[B84] Young L. E. Sinclair K. D. Wilmut I. (1998). Large offspring syndrome in cattle and sheep. *Rev. Reprod.* 3 155–163. 9829550 10.1530/ror.0.0030155

[B85] Zaitoun I. Downs K. M. Rosa G. J. M. Khatib H. (2010). Upregulation of imprinted genes in mice. *Epigenetics* 5 149–158. 20168089 10.4161/epi.5.2.11081 PMC3020655

[B86] Zhu P. Guo H. Ren Y. Hou Y. Dong J. Li R. (2018). Single-cell DNA methylome sequencing of human preimplantation embryos. *Nat. Genet.* 50 12–19. 10.1038/s41588-017-0007-6 29255258

[B87] Zilberman D. (2017). An evolutionary case for functional gene body methylation in plants and animals. *Genome Biol.* 18:87. 10.1186/s13059-017-1230-2 28486944 PMC5423413

