# Supplementary material for: Methylome Dynamics of Bovine Gametes and in vivo Early Embryos
Source: Front Genet. 2019 May 28;10:512. doi: 10.3389/fgene.2019.00512 (PMC6546829; doi:10.3389/fgene.2019.00512)
Supplement: Supplementary file 6 [file Presentation_1.PPTX]

## Slide 1
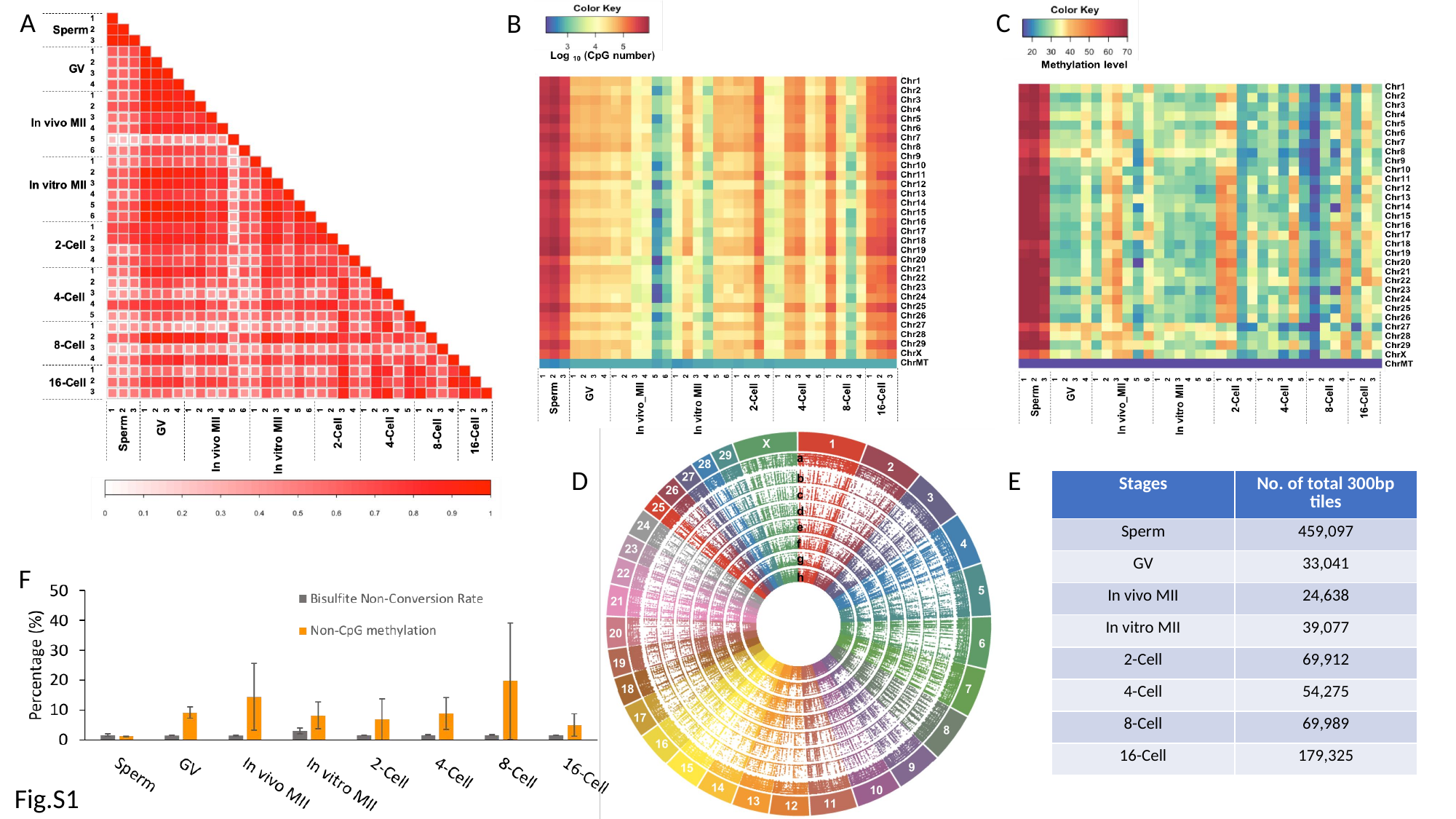

A
C
B
D
E
| Stages | No. of total 300bp tiles |
| --- | --- |
| Sperm | 459,097 |
| GV | 33,041 |
| In vivo MII | 24,638 |
| In vitro MII | 39,077 |
| 2-Cell | 69,912 |
| 4-Cell | 54,275 |
| 8-Cell | 69,989 |
| 16-Cell | 179,325 |
F
Fig.S1

## Slide 2
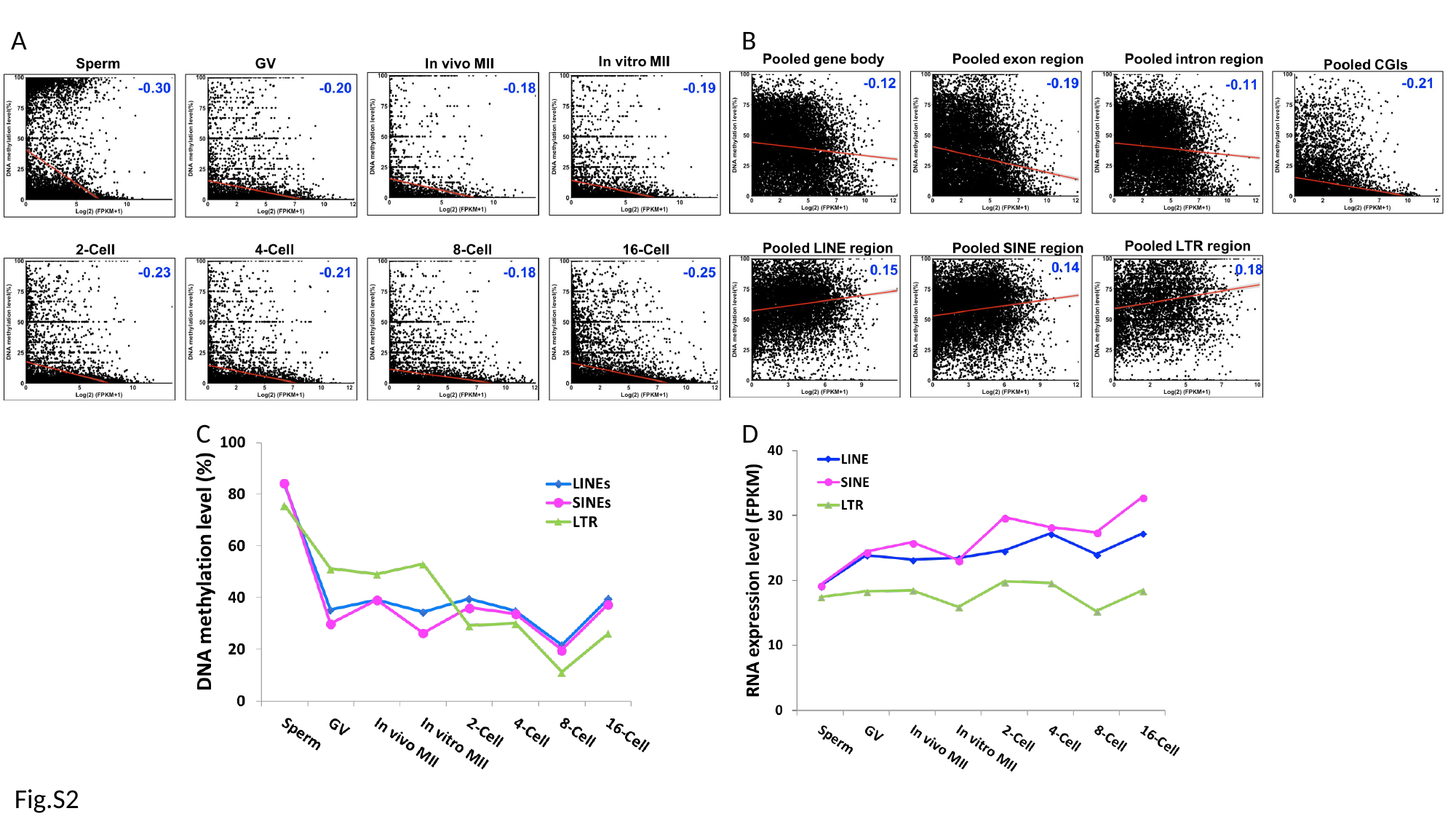

A
B
C
D
Fig.S2

## Slide 3
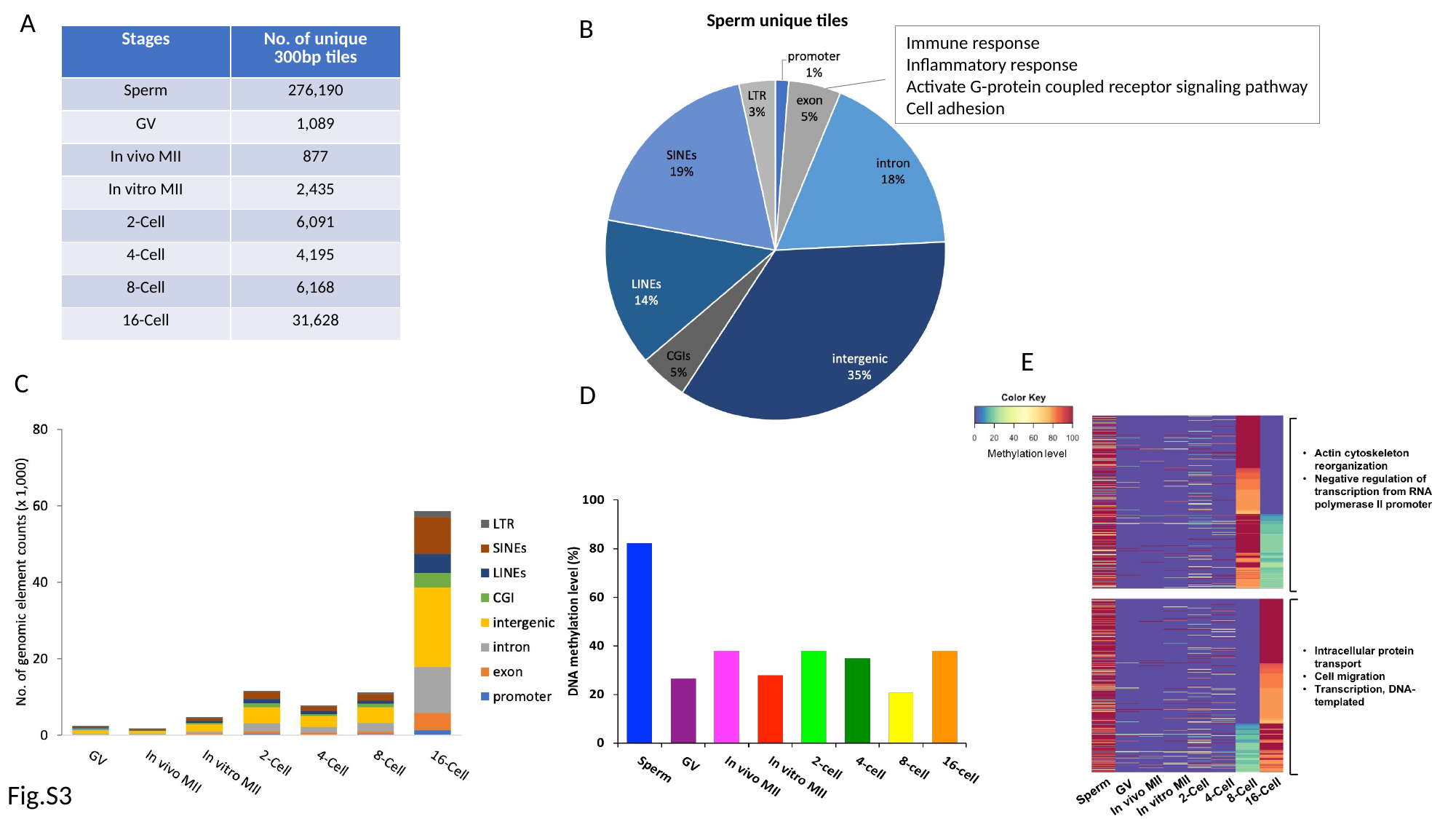

A
Sperm unique tiles
B
| Stages | No. of unique 300bp tiles |
| --- | --- |
| Sperm | 276,190 |
| GV | 1,089 |
| In vivo MII | 877 |
| In vitro MII | 2,435 |
| 2-Cell | 6,091 |
| 4-Cell | 4,195 |
| 8-Cell | 6,168 |
| 16-Cell | 31,628 |
Immune response
Inflammatory response
Activate G-protein coupled receptor signaling pathway
Cell adhesion
E
C
D
Fig.S3

## Slide 4
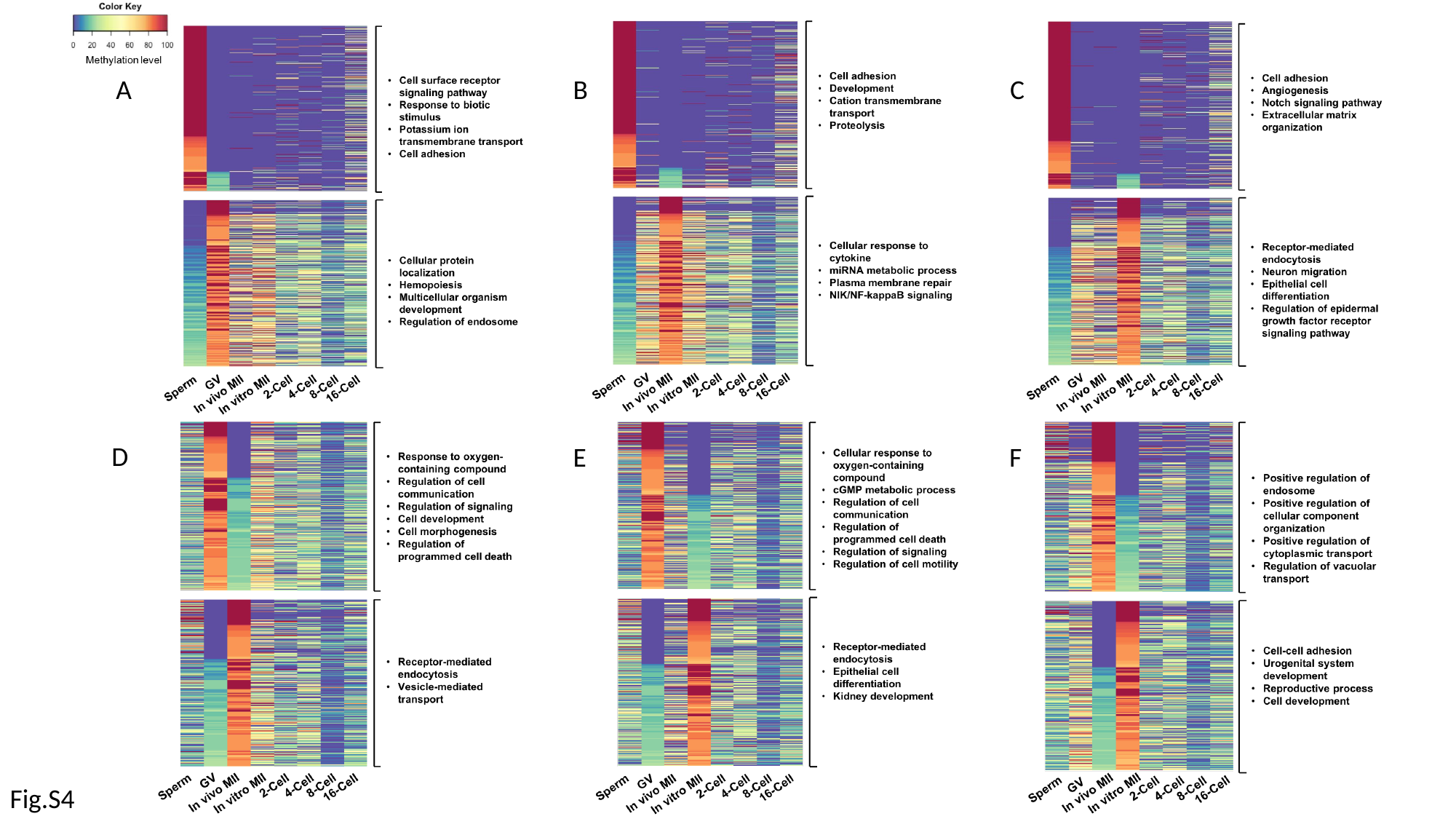

A
B
C
D
E
F
Fig.S4
