# Supplementary material for: Methylome Dynamics of Bovine Gametes and in vivo Early Embryos
Source: Front Genet. 2019 May 28;10:512. doi: 10.3389/fgene.2019.00512 (PMC6546829; doi:10.3389/fgene.2019.00512)
Supplement: Supplementary file 7 [file Data_Sheet_1.pdf]

Supplementary figure and table legend

Figure S1. Methylome profiles of bovine gametes and in vivo developed embryos
The Pearson correlation heatmap (A) between stages. Color key: white low correlation, red high correlation. Heatmaps of the numbers (Color key: purple: low CpG number, red high CpG numbers) (B) and methylation levels (Color key: purple: hypomethylation, red hypermethylation) (C) of captured CpGs in each chromosome. Circos plot (D) visualization of all methylated 300-bp tiles of each stage of pre-implantation embryonic development. a. sperm, b. GV oocytes, c. *in vivo* MII oocytes, d. *in vitro* MII oocytes, e. 2-cell, f. 4-cell, g. 8-cell, h. 16-cell. The number (E) of total captured 300-bp tiles in each stage. Bar plot (F) of non-CpG methylation level and bisulfite non-conversion rate of each stage. GV: germinal vesicle oocytes; MII: matured oocytes.

Figure S2. Relationship between transcriptomes and methylomes of bovine gametes and in vivo developed embryos
Correlation between gene expression and methylation of the promoters (A), gene bodies, exons, introns, CGIs (B). Line plots of DNA methylation (C) and RNA expression levels (D) of repetitive elements LINEs, SINEs, and LTR in each development stage. GV: germinal vesicle oocytes; MII: matured oocytes.

Figure S3. Uniquely methylated regions in bovine gametes and in vivo developed embryos
The numbers (A) of uniquely methylated 300-bp tiles in each development stage. Pie plot (B) of the distribution of uniquely methylated tiles in sperm categorized by genomic regions and the associated GO terms. Stack bar plot (C) of uniquely methylated 300-bp tiles in each stage categorized by genomic regions. Bar plot (D) of averaged DNA methylation levels in uniquely methylated regions across stages. Heatmap (E) of DMRs between the 8- and 16-cell stages that were hypermethylated in the 8-cell (upper) or 16-cell (lower) and their GO term representatives. GV: germinal vesicle oocytes; MII: matured oocytes. (Color key: purple: hypomethylation, red hypermethylation).

Figure S4. DMRs between different types of gametes and their GO term representatives.
Heatmaps of DMRs: sperm vs. GV (A), sperm vs. *in vivo* MII (B), sperm vs. *in vitro* MII (C), GV vs. *in vivo* MII (D), GV vs. *in vitro* MII (E), and *in vivo* MII vs. *in vitro* MII (F). GV: germinal vesicle oocytes; MII: matured oocytes. (Color key: purple: hypomethylation, red hypermethylation)

Table S1. Summary of WGBS library mapping and data processing

Table S2.1. Gene Ontology (GO) terms for genes with commonly methylated introns among all samples **Table S2.2.** Genes with commonly methylated introns among all samples

Table S3. Gene Ontology (GO) terms for hypermethylated genes in sperm (Table S3.1), GV (Table S3.3), *in vivo* MII (Table S3.5), *in vitro* MII (Table S3.7), 2-cell stage (Table S3.9), 4-cell stage (Table S3.11), 8-cell stage (Table S3.13), and 16-cell stage (Table S3.15). Gene list of hypermethylated genes in sperm (Table S3.2), GV (Table S3.4), *in vivo* MII (Table S3.6), *in vitro* MII (Table S3.8), 2-cell stage (Table S3.10), 4-cell stage (Table S3.12), 8-cell stage (Table S3.14), and 16-cell stage (Table S3.16)

Table S4. GO terms of DMRs between 8 vs. 16-cell that hypermethylated in 8-cell (Table S4.1) or 16-cell (Table S4.3). Genes in DMRs between the 8- and 16-cell embryos that are hypermethylated in the 8-cell embryos (Table S4.2) or 16-cell (Table S4.4)

Table S5. GO terms of DMRs between sperm vs. GV (Table S5.1), sperm vs. *in vivo* MII (Table S5.3), sperm vs. *in vitro* MII (Table S5.5), GV vs. *in vivo* MII (Table S5.7), GV vs. *in vitro* MII (Table S5.9), and *in vivo* MII vs. *in vitro* MII (Table S5.11). Genes in DMRs between sperm vs. GV (Table S5.2), sperm vs. *in vivo* MII (Table S5.4), sperm vs. *in vitro* MII (Table S5.6), GV vs. *in vivo* MII (Table S5.8), GV vs. *in vitro* MII (Table S5.10), and *in vivo* MII vs. *in vitro* MII (Table S5.12)
